# *N*-(2-Nitro­phen­yl)thio­phene-2-carbox­amide

**DOI:** 10.1107/S160053681400912X

**Published:** 2014-04-26

**Authors:** Rodolfo Moreno-Fuquen, Alexis Azcárate, Alan R. Kennedy

**Affiliations:** a Departamento de Química - Facultad de Ciencias, Universidad del Valle, Apartado 25360, Santiago de Cali, Colombia; b WestCHEM, Department of Pure and Applied Chemistry, University of Strathclyde, 295 Cathedral Street, Glasgow G1 1XL, Scotland

## Abstract

The title compound, C_11_H_8_N_2_O_3_S, shows two mol­ecules per asymmetric unit, with the dihedral angles between the benzene and thio­phene rings of 13.53 (6) and 8.50 (5)° being a notable difference between them. An intra­molecular N—H⋯O hydrogen-bond in each mol­ecule generates an *S*(6) ring motif. The crystal packing shows no classical hydrogen bonds with the mol­ecules being packed to form weak C—H⋯O and C—H⋯S inter­actions leading to *R*_2_^2^(9) and *R*_4_^4^(25) rings which are edge-shared, giving layers parallel to (010).

## Related literature   

For the anti­bacterial and anti­fungal activity of amide compounds, see: Aytemir *et al.* (2003 ▶); Hrelia *et al.* (1995 ▶). For a similar compound, see: Moreno-Fuquen *et al.* (2013 ▶). For hydrogen-bonding information, see: Nardelli (1995 ▶). For hydrogen-bond motifs, see: Etter *et al.* (1990 ▶).
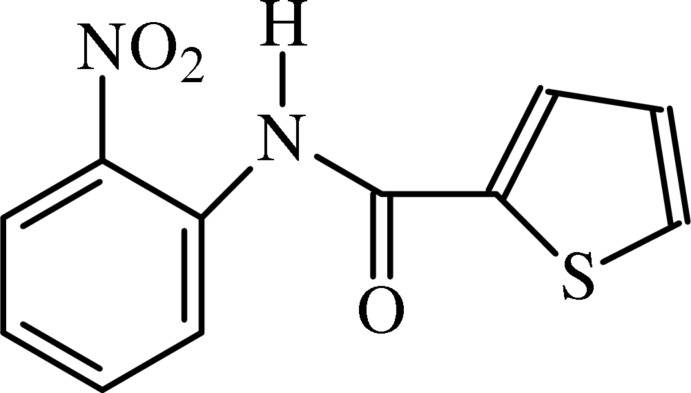


## Experimental   

### 

#### Crystal data  

C_11_H_8_N_2_O_3_S
*M* *_r_* = 248.25
Monoclinic, 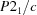

*a* = 11.6359 (3) Å
*b* = 13.2501 (3) Å
*c* = 17.7412 (4) Å
β = 129.898 (1)°
*V* = 2098.47 (9) Å^3^
*Z* = 8
Mo *K*α radiation
μ = 0.31 mm^−1^
*T* = 123 K
0.28 × 0.15 × 0.05 mm

#### Data collection  

Oxford Diffraction Xcalibur E diffractometer
Absorption correction: multi-scan (*CrysAlis PRO*; Oxford Diffraction, 2010 ▶) *T*_min_ = 0.953, *T*_max_ = 1.000
10418 measured reflections
5093 independent reflections
4012 reflections with *I* > 2σ(*I*)
*R*_int_ = 0.028

#### Refinement  

*R*[*F* ^2^ > 2σ(*F* ^2^)] = 0.047
*wR*(*F* ^2^) = 0.116
*S* = 1.04
5093 reflections
315 parameters
H atoms treated by a mixture of independent and constrained refinement
Δρ_max_ = 0.49 e Å^−3^
Δρ_min_ = −0.43 e Å^−3^

### 

Data collection: *CrysAlis PRO* (Oxford Diffraction, 2010 ▶); cell refinement: *CrysAlis PRO*; data reduction: *CrysAlis PRO*; program(s) used to solve structure: *SHELXS97* (Sheldrick, 2008 ▶); program(s) used to refine structure: *SHELXL97* (Sheldrick, 2008 ▶); molecular graphics: *ORTEP-3 for Windows* (Farrugia, 2012 ▶) and *Mercury* (Macrae *et al.*, 2006 ▶); software used to prepare material for publication: *WinGX* (Farrugia, 2012 ▶).

## Supplementary Material

Crystal structure: contains datablock(s) I, global. DOI: 10.1107/S160053681400912X/tk5309sup1.cif

Structure factors: contains datablock(s) I. DOI: 10.1107/S160053681400912X/tk5309Isup2.hkl

Supporting information file. DOI: 10.1107/S160053681400912X/tk5309Isup3.cml

CCDC reference: 998902

Additional supporting information:  crystallographic information; 3D view; checkCIF report

## Figures and Tables

**Table 1:** Hydrogen-bond geometry (Å, °)

*D*—H⋯*A*	*D*—H	H⋯*A*	*D*⋯*A*	*D*—H⋯*A*
N1—H1*N*⋯O2	0.81 (3)	1.96 (3)	2.634 (2)	140 (3)
N3—H3*N*⋯O5	0.84 (2)	1.93 (2)	2.616 (2)	138 (2)
C1—H1⋯O4^i^	0.95	2.50	3.209 (3)	131
C8—H8⋯O5^ii^	0.95	2.52	3.248 (3)	133
C19—H19⋯O2^iii^	0.95	2.63	3.377 (3)	136
C11—H11⋯S2^iii^	0.95	2.92	3.732 (2)	144
C22—H22⋯S1^ii^	0.95	2.84	3.689 (2)	149
C12—H12⋯O1^iv^	0.95	2.47	3.198 (3)	133

Symmetry codes: (i) 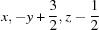; (ii) 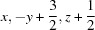; (iii) 
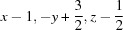
; (iv) 
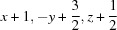
.

**Fig. 1.**
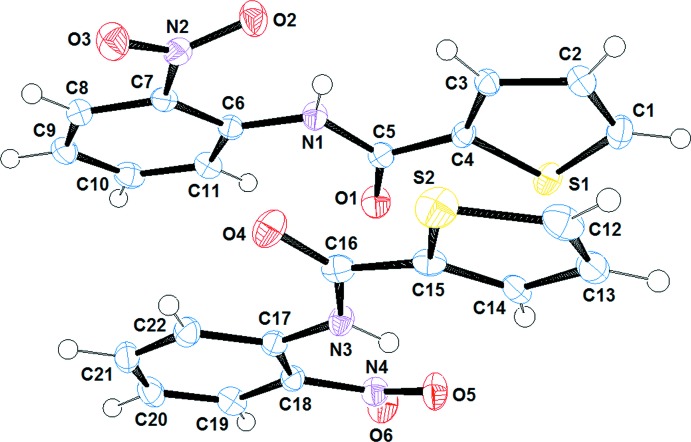
Molecular conformation and atom numbering scheme for the title compound with displacement ellipsoids drawn at the 50% probability level. H atoms are shown as spheres of arbitrary radius.

**Fig. 2.**
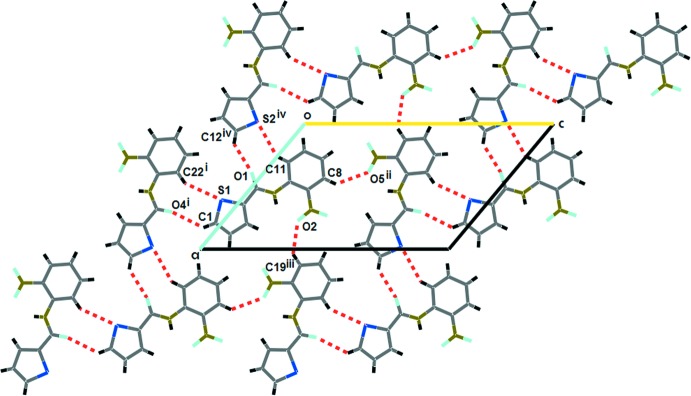
Part of the crystal structure of (I), showing the formation of edge-fused R22(9) and R44(25) rings running parallel to (010).

## supplementary crystallographic information

### 2.1. Synthesis and crystallization

The reagents and solvents for the synthesis were obtained from the Aldrich Chemical Co., and were used without additional purification. The title molecule was synthesized using equimolar qu­anti­ties of 2-thio­phene­carbonyl chloride (0.376 g., 2.565 mmol) and 2-nitro­aniline (0.354 g). The reagents were dissolved in 10 mL of aceto­nitrile and the solution was taken to reflux in constant stirring for 1 hour. Yellow crystals of good quality were obtained after leaving the solvent to evaporate. M.pt: 397 (1) K.

### 2.2. Refinement

All H-atoms were positioned at geometrically idealized positions with a C—H distance of 0.95 Å and *U*_iso_(H) = 1.2*U*_eq_ of the atoms to which they were bonded. The H1N and H3N atoms were found from Fourier maps and were refined freely.

### 3. Results and discussion

The present compound forms part of a systematic work on N-aromatic amides in our research group. Anti­bacterial and anti­fungal activities of different carb­oxy­amide derivatives have been reported (Aytemir *et al.*, 2003). Derivatives of thio­phene carboxanilide compounds present genotoxicity in bacterial mammalian and human cells (Hrelia *et al.*, 1995). In the synthesis of the amides, the 2-nitro­aniline is taken as a template, in order to study the structural changes and the supra­molecular behavior by the reaction of different ligands with this precursor (Moreno-Fuquen *et al.*, 2013). With this aim, the synthesis of N-(2-nitro­phenyl)­thio­phene-2-carboxamide (I) was undertaken. The structure of N-(2-nitro­phenyl)­furan-2-carboxamide (2NPFC), (Moreno-Fuquen *et al.*, 2013), a similar compound, was taken to compare with the structural parameters of (I).

The title compound shows two molecules (A and B) per asymmetric unit (see Fig. 1). Compound (I) exhibits dihedral angles between the benzene and thio­phene rings of 13.53 (6)° and 8.50 (5)° for A and B, respectively. These dihedral angles are very similar to the value presented in the (2NPFC) system [9.71 (5)°]. The other bond lengths and bond angles agree closely with those values presented in its homologous amide (2NPFC), except for the C—S distances, which for obvious reasons are different on the furan rings. The nitro groups form dihedral angles with the adjacent benzene ring of 15.44 (4) and 16.07 (6)° for O2—N2—O3 and O5—N4—O6, respectively.

The crystal packing shows no classical hydrogen bonds and the molecules are packed forming weak C—H···O and C—H···S inter­actions and they are propagated parallel to (010) (see Fig. 2). According to the graph-set assignment, the intra­molecular hydrogen-bond pattern generates a S(6) ring motif (Etter, 1990) (see Table 1). The C8 and C11 atoms of the phenyl ring and C1 atom of the thio­phene ring at (*x*, *y*, *z*), act as hydrogen-bond donors to the nitro O5 atom at (*x*, -*y*+3/2, +*z*+1/2), to the S2 atom at (*x*-1, -*y*+3/2, +*z*-1/2) and to the carbonyl atom O4 at (*x*, -*y*+3/2, +*z*-1/2), respectively. Additionally, the C19 and C22 atoms of the benzene ring and C12 atom of the thio­phene ring at (*x*, *y*, *z*) act as hydrogen-bond donors to the nitro O2 atom at (*x*-1, -*y*+3/2, +*z*-1/2), to the S1 atom at (*x*, -*y*+3/2, +*z*+1/2) and to the carbonyl O1 atom at (*x*+1, -*y*+3/2, +*z*+1/2), respectively, (see Table 1; Nardelli, 1995). All these inter­actions form an *R*^2^_2_(9) and *R*^4^_4_(25) edge-fused ring which are running parallel to (010) (see Fig. 2).

### Crystal data

**Table:**

C_11_H_8_N_2_O_3_S	*F*(000) = 1024
*M**_r_* = 248.25	*D*_x_ = 1.572 Mg m^−^^3^
Monoclinic, *P*2_1_/*c*	Melting point: 397(1) K
Hall symbol: -P 2ybc	Mo *K*α radiation, λ = 0.71073 Å
*a* = 11.6359 (3) Å	Cell parameters from 4181 reflections
*b* = 13.2501 (3) Å	θ = 3.4–29.4°
*c* = 17.7412 (4) Å	µ = 0.31 mm^−^^1^
β = 129.898 (1)°	*T* = 123 K
*V* = 2098.47 (9) Å^3^	Tablet, yellow
*Z* = 8	0.28 × 0.15 × 0.05 mm

### Data collection

**Table:**

Oxford Diffraction Xcalibur E diffractometer	5093 independent reflections
Radiation source: fine-focus sealed tube	4012 reflections with *I* > 2σ(*I*)
Graphite monochromator	*R*_int_ = 0.028
ω scans	θ_max_ = 29.4°, θ_min_ = 3.4°
Absorption correction: multi-scan (*CrysAlis PRO*; Oxford Diffraction, 2010)	*h* = −15→14
*T*_min_ = 0.953, *T*_max_ = 1.000	*k* = −15→17
10418 measured reflections	*l* = −22→24

### Refinement

**Table:**

Refinement on *F*^2^	Primary atom site location: structure-invariant direct methods
Least-squares matrix: full	Secondary atom site location: difference Fourier map
*R*[*F*^2^ > 2σ(*F*^2^)] = 0.047	Hydrogen site location: inferred from neighbouring sites
*wR*(*F*^2^) = 0.116	H atoms treated by a mixture of independent and constrained refinement
*S* = 1.04	*w* = 1/[σ^2^(*F*_o_^2^) + (0.042*P*)^2^ + 1.7613*P*] where *P* = (*F*_o_^2^ + 2*F*_c_^2^)/3
5093 reflections	(Δ/σ)_max_ < 0.001
315 parameters	Δρ_max_ = 0.49 e Å^−^^3^
0 restraints	Δρ_min_ = −0.43 e Å^−^^3^

### Special details

**Table:**

Geometry. All esds (except the esd in the dihedral angle between two l.s. planes) are estimated using the full covariance matrix. The cell esds are taken into account individually in the estimation of esds in distances, angles and torsion angles; correlations between esds in cell parameters are only used when they are defined by crystal symmetry. An approximate (isotropic) treatment of cell esds is used for estimating esds involving l.s. planes.
Refinement. Refinement of *F*^2^ against ALL reflections. The weighted *R*-factor *wR* and goodness of fit *S* are based on *F*^2^, conventional *R*-factors *R* are based on *F*, with *F* set to zero for negative *F*^2^. The threshold expression of *F*^2^ > σ(*F*^2^) is used only for calculating *R*-factors(gt) *etc*. and is not relevant to the choice of reflections for refinement. *R*-factors based on *F*^2^ are statistically about twice as large as those based on *F*, and *R*- factors based on ALL data will be even larger.

### Fractional atomic coordinates and isotropic or equivalent isotropic displacement parameters (Å^2^)

**Table:**

	*x*	*y*	*z*	*U*_iso_*/*U*_eq_	
S1	0.60696 (6)	0.81889 (4)	−0.07981 (4)	0.02441 (14)	
S2	0.98321 (6)	0.64159 (5)	0.30229 (4)	0.03015 (16)	
O1	0.38729 (16)	0.85064 (13)	−0.05031 (11)	0.0255 (4)	
O2	0.77371 (16)	0.93931 (13)	0.29784 (11)	0.0262 (4)	
O3	0.73314 (17)	0.91094 (13)	0.39862 (11)	0.0274 (4)	
O4	0.70355 (17)	0.70186 (12)	0.26329 (11)	0.0269 (4)	
O5	0.38579 (16)	0.59679 (13)	−0.08094 (11)	0.0268 (4)	
O6	0.15254 (17)	0.63885 (14)	−0.18258 (11)	0.0303 (4)	
N1	0.5542 (2)	0.90436 (14)	0.10981 (13)	0.0184 (4)	
N2	0.68724 (19)	0.92377 (13)	0.31503 (12)	0.0193 (4)	
N3	0.54164 (19)	0.64179 (14)	0.10488 (13)	0.0189 (4)	
N4	0.26905 (19)	0.62585 (14)	−0.09860 (13)	0.0217 (4)	
C1	0.7915 (3)	0.82021 (17)	−0.02508 (16)	0.0247 (5)	
H1	0.8288	0.8029	−0.0578	0.030*	
C2	0.8804 (2)	0.84884 (17)	0.07096 (16)	0.0220 (4)	
H2	0.9864	0.8533	0.1126	0.026*	
C3	0.7965 (2)	0.87137 (16)	0.10222 (15)	0.0182 (4)	
H3	0.8395	0.8922	0.1667	0.022*	
C4	0.6440 (2)	0.85876 (15)	0.02591 (14)	0.0171 (4)	
C5	0.5153 (2)	0.87066 (16)	0.02341 (14)	0.0180 (4)	
C6	0.4632 (2)	0.91537 (15)	0.13495 (14)	0.0171 (4)	
C7	0.5258 (2)	0.92336 (15)	0.23390 (15)	0.0173 (4)	
C8	0.4367 (2)	0.93183 (16)	0.26042 (16)	0.0218 (4)	
H8	0.4816	0.9355	0.3278	0.026*	
C9	0.2828 (2)	0.93496 (17)	0.18862 (17)	0.0240 (5)	
H9	0.2211	0.9416	0.2060	0.029*	
C10	0.2195 (2)	0.92823 (17)	0.09095 (16)	0.0230 (5)	
H10	0.1136	0.9307	0.0414	0.028*	
C11	0.3066 (2)	0.91801 (16)	0.06396 (15)	0.0200 (4)	
H11	0.2599	0.9127	−0.0036	0.024*	
C12	1.0568 (2)	0.60694 (18)	0.24879 (17)	0.0268 (5)	
H12	1.1610	0.5988	0.2838	0.032*	
C13	0.9500 (2)	0.59212 (17)	0.15022 (16)	0.0230 (5)	
H13	0.9720	0.5724	0.1092	0.028*	
C14	0.8015 (2)	0.60940 (16)	0.11495 (15)	0.0185 (4)	
H14	0.7132	0.6030	0.0483	0.022*	
C15	0.8039 (2)	0.63702 (16)	0.19220 (14)	0.0188 (4)	
C16	0.6807 (2)	0.66372 (16)	0.19215 (15)	0.0194 (4)	
C17	0.4018 (2)	0.65097 (15)	0.08063 (14)	0.0172 (4)	
C18	0.2688 (2)	0.64194 (16)	−0.01743 (15)	0.0182 (4)	
C19	0.1290 (2)	0.64708 (17)	−0.04208 (16)	0.0232 (5)	
H19	0.0408	0.6408	−0.1088	0.028*	
C20	0.1180 (3)	0.66121 (17)	0.03006 (18)	0.0256 (5)	
H20	0.0227	0.6636	0.0137	0.031*	
C21	0.2479 (3)	0.67189 (17)	0.12687 (17)	0.0240 (5)	
H21	0.2409	0.6827	0.1767	0.029*	
C22	0.3873 (2)	0.66709 (16)	0.15197 (16)	0.0212 (4)	
H22	0.4747	0.6749	0.2187	0.025*	
H3N	0.536 (3)	0.6175 (18)	0.0592 (18)	0.020 (6)*	
H1N	0.642 (3)	0.914 (2)	0.156 (2)	0.033 (7)*	

### Atomic displacement parameters (Å^2^)

**Table:**

	*U*^11^	*U*^22^	*U*^33^	*U*^12^	*U*^13^	*U*^23^
S1	0.0264 (3)	0.0289 (3)	0.0169 (2)	0.0012 (2)	0.0134 (2)	−0.0021 (2)
S2	0.0209 (3)	0.0372 (4)	0.0198 (3)	−0.0010 (2)	0.0073 (2)	−0.0030 (2)
O1	0.0176 (7)	0.0359 (9)	0.0171 (7)	−0.0009 (7)	0.0085 (6)	−0.0018 (6)
O2	0.0178 (7)	0.0417 (10)	0.0203 (7)	−0.0054 (7)	0.0129 (6)	−0.0059 (7)
O3	0.0270 (8)	0.0359 (9)	0.0166 (7)	−0.0021 (7)	0.0128 (7)	0.0020 (7)
O4	0.0264 (8)	0.0321 (9)	0.0173 (7)	0.0015 (7)	0.0118 (6)	−0.0039 (7)
O5	0.0185 (7)	0.0425 (10)	0.0209 (7)	−0.0019 (7)	0.0134 (6)	−0.0061 (7)
O6	0.0232 (8)	0.0442 (10)	0.0150 (7)	0.0044 (7)	0.0084 (6)	0.0006 (7)
N1	0.0128 (8)	0.0254 (10)	0.0147 (8)	−0.0009 (7)	0.0077 (7)	−0.0020 (7)
N2	0.0199 (8)	0.0193 (9)	0.0179 (8)	−0.0012 (7)	0.0117 (7)	−0.0020 (7)
N3	0.0182 (8)	0.0236 (9)	0.0138 (8)	−0.0002 (7)	0.0098 (7)	−0.0025 (7)
N4	0.0191 (9)	0.0257 (10)	0.0179 (8)	−0.0016 (8)	0.0109 (7)	−0.0025 (7)
C1	0.0308 (12)	0.0253 (11)	0.0261 (11)	0.0032 (10)	0.0220 (10)	0.0014 (9)
C2	0.0204 (10)	0.0241 (11)	0.0230 (10)	−0.0010 (9)	0.0147 (9)	0.0006 (9)
C3	0.0210 (10)	0.0174 (10)	0.0203 (10)	−0.0003 (8)	0.0150 (9)	0.0010 (8)
C4	0.0213 (10)	0.0165 (10)	0.0148 (9)	0.0010 (8)	0.0122 (8)	0.0008 (7)
C5	0.0205 (10)	0.0170 (10)	0.0159 (9)	0.0011 (8)	0.0114 (8)	0.0024 (8)
C6	0.0191 (10)	0.0150 (10)	0.0183 (9)	0.0004 (8)	0.0125 (8)	0.0012 (8)
C7	0.0166 (9)	0.0154 (10)	0.0191 (9)	−0.0010 (8)	0.0112 (8)	−0.0005 (8)
C8	0.0268 (11)	0.0205 (11)	0.0243 (10)	−0.0044 (9)	0.0191 (9)	−0.0035 (8)
C9	0.0243 (11)	0.0228 (11)	0.0336 (12)	−0.0039 (9)	0.0225 (10)	−0.0041 (9)
C10	0.0167 (10)	0.0220 (11)	0.0268 (11)	−0.0008 (9)	0.0124 (9)	0.0003 (9)
C11	0.0178 (10)	0.0200 (10)	0.0192 (10)	0.0012 (8)	0.0106 (8)	0.0026 (8)
C12	0.0165 (10)	0.0279 (12)	0.0274 (11)	−0.0012 (9)	0.0102 (9)	0.0028 (9)
C13	0.0225 (10)	0.0215 (11)	0.0275 (11)	−0.0006 (9)	0.0172 (9)	0.0016 (9)
C14	0.0160 (9)	0.0175 (10)	0.0232 (10)	0.0005 (8)	0.0131 (8)	0.0041 (8)
C15	0.0168 (9)	0.0171 (10)	0.0157 (9)	−0.0015 (8)	0.0073 (8)	0.0006 (8)
C16	0.0213 (10)	0.0162 (10)	0.0156 (9)	0.0012 (8)	0.0095 (8)	0.0033 (8)
C17	0.0186 (10)	0.0158 (10)	0.0174 (9)	0.0009 (8)	0.0117 (8)	0.0015 (8)
C18	0.0214 (10)	0.0172 (10)	0.0175 (9)	0.0006 (8)	0.0132 (8)	0.0008 (8)
C19	0.0203 (10)	0.0234 (11)	0.0233 (10)	0.0007 (9)	0.0128 (9)	0.0026 (9)
C20	0.0254 (11)	0.0236 (11)	0.0375 (13)	0.0031 (9)	0.0246 (10)	0.0047 (10)
C21	0.0334 (12)	0.0210 (11)	0.0293 (11)	0.0036 (9)	0.0255 (10)	0.0033 (9)
C22	0.0265 (11)	0.0202 (11)	0.0193 (10)	0.0021 (9)	0.0158 (9)	0.0022 (8)

### Geometric parameters (Å, º)

**Table:**

S1—C1	1.697 (2)	C6—C11	1.401 (3)
S1—C4	1.716 (2)	C6—C7	1.408 (3)
S2—C12	1.699 (3)	C7—C8	1.391 (3)
S2—C15	1.716 (2)	C8—C9	1.380 (3)
O1—C5	1.225 (2)	C8—H8	0.9500
O2—N2	1.242 (2)	C9—C10	1.384 (3)
O3—N2	1.224 (2)	C9—H9	0.9500
O4—C16	1.220 (3)	C10—C11	1.376 (3)
O5—N4	1.243 (2)	C10—H10	0.9500
O6—N4	1.223 (2)	C11—H11	0.9500
N1—C5	1.366 (3)	C12—C13	1.361 (3)
N1—C6	1.395 (3)	C12—H12	0.9500
N1—H1N	0.81 (3)	C13—C14	1.430 (3)
N2—O2	1.242 (2)	C13—H13	0.9500
N2—C7	1.460 (3)	C14—C15	1.401 (3)
N3—C16	1.373 (3)	C14—H14	0.9500
N3—C17	1.396 (3)	C15—C16	1.476 (3)
N3—H3N	0.84 (2)	C17—C22	1.396 (3)
N4—O5	1.243 (2)	C17—C18	1.408 (3)
N4—C18	1.457 (3)	C18—C19	1.390 (3)
C1—C2	1.362 (3)	C19—C20	1.378 (3)
C1—H1	0.9500	C19—H19	0.9500
C2—C3	1.429 (3)	C20—C21	1.387 (3)
C2—H2	0.9500	C20—H20	0.9500
C3—C4	1.388 (3)	C21—C22	1.381 (3)
C3—H3	0.9500	C21—H21	0.9500
C4—C5	1.478 (3)	C22—H22	0.9500
			
C1—S1—C4	91.88 (10)	C8—C9—C10	119.2 (2)
C12—S2—C15	92.00 (11)	C8—C9—H9	120.4
C5—N1—C6	128.35 (18)	C10—C9—H9	120.4
C5—N1—H1N	118.8 (19)	C11—C10—C9	121.5 (2)
C6—N1—H1N	112.4 (19)	C11—C10—H10	119.3
O3—N2—O2	121.89 (17)	C9—C10—H10	119.3
O3—N2—O2	121.89 (17)	C10—C11—C6	120.8 (2)
O3—N2—C7	118.65 (17)	C10—C11—H11	119.6
O2—N2—C7	119.44 (17)	C6—C11—H11	119.6
O2—N2—C7	119.44 (17)	C13—C12—S2	112.66 (17)
C16—N3—C17	128.81 (18)	C13—C12—H12	123.7
C16—N3—H3N	118.3 (16)	S2—C12—H12	123.7
C17—N3—H3N	112.9 (16)	C12—C13—C14	112.9 (2)
O6—N4—O5	121.92 (18)	C12—C13—H13	123.5
O6—N4—O5	121.92 (18)	C14—C13—H13	123.5
O6—N4—C18	118.68 (18)	C15—C14—C13	110.71 (18)
O5—N4—C18	119.36 (17)	C15—C14—H14	124.6
O5—N4—C18	119.36 (17)	C13—C14—H14	124.6
C2—C1—S1	112.67 (17)	C14—C15—C16	130.68 (18)
C2—C1—H1	123.7	C14—C15—S2	111.69 (16)
S1—C1—H1	123.7	C16—C15—S2	117.62 (15)
C1—C2—C3	112.57 (19)	O4—C16—N3	124.8 (2)
C1—C2—H2	123.7	O4—C16—C15	122.12 (19)
C3—C2—H2	123.7	N3—C16—C15	113.07 (18)
C4—C3—C2	111.11 (18)	C22—C17—N3	121.89 (18)
C4—C3—H3	124.4	C22—C17—C18	117.21 (19)
C2—C3—H3	124.4	N3—C17—C18	120.89 (18)
C3—C4—C5	130.78 (18)	C19—C18—C17	121.45 (19)
C3—C4—S1	111.76 (15)	C19—C18—N4	116.11 (18)
C5—C4—S1	117.42 (15)	C17—C18—N4	122.44 (18)
O1—C5—N1	124.7 (2)	C20—C19—C18	120.1 (2)
O1—C5—C4	121.59 (19)	C20—C19—H19	119.9
N1—C5—C4	113.69 (17)	C18—C19—H19	119.9
N1—C6—C11	122.09 (18)	C19—C20—C21	119.1 (2)
N1—C6—C7	120.96 (18)	C19—C20—H20	120.4
C11—C6—C7	116.95 (18)	C21—C20—H20	120.4
C8—C7—C6	121.73 (19)	C22—C21—C20	121.1 (2)
C8—C7—N2	115.67 (18)	C22—C21—H21	119.4
C6—C7—N2	122.60 (18)	C20—C21—H21	119.4
C9—C8—C7	119.8 (2)	C21—C22—C17	121.0 (2)
C9—C8—H8	120.1	C21—C22—H22	119.5
C7—C8—H8	120.1	C17—C22—H22	119.5
			
O2—O2—N2—O3	0.0 (5)	N1—C6—C11—C10	179.5 (2)
O2—O2—N2—C7	0.0 (5)	C7—C6—C11—C10	0.1 (3)
O5—O5—N4—O6	0.00 (11)	C15—S2—C12—C13	0.07 (19)
O5—O5—N4—C18	0.00 (6)	S2—C12—C13—C14	0.1 (3)
C4—S1—C1—C2	0.65 (18)	C12—C13—C14—C15	−0.4 (3)
S1—C1—C2—C3	−0.3 (3)	C13—C14—C15—C16	178.9 (2)
C1—C2—C3—C4	−0.3 (3)	C13—C14—C15—S2	0.4 (2)
C2—C3—C4—C5	178.5 (2)	C12—S2—C15—C14	−0.27 (17)
C2—C3—C4—S1	0.8 (2)	C12—S2—C15—C16	−178.96 (17)
C1—S1—C4—C3	−0.82 (17)	C17—N3—C16—O4	−4.7 (4)
C1—S1—C4—C5	−178.85 (17)	C17—N3—C16—C15	175.74 (19)
C6—N1—C5—O1	5.4 (4)	C14—C15—C16—O4	−167.8 (2)
C6—N1—C5—C4	−174.01 (19)	S2—C15—C16—O4	10.6 (3)
C3—C4—C5—O1	−175.9 (2)	C14—C15—C16—N3	11.8 (3)
S1—C4—C5—O1	1.7 (3)	S2—C15—C16—N3	−169.80 (15)
C3—C4—C5—N1	3.6 (3)	C16—N3—C17—C22	−12.8 (3)
S1—C4—C5—N1	−178.86 (15)	C16—N3—C17—C18	168.7 (2)
C5—N1—C6—C11	−17.6 (3)	C22—C17—C18—C19	−1.1 (3)
C5—N1—C6—C7	161.8 (2)	N3—C17—C18—C19	177.51 (19)
N1—C6—C7—C8	−178.37 (19)	C22—C17—C18—N4	179.48 (19)
C11—C6—C7—C8	1.0 (3)	N3—C17—C18—N4	−1.9 (3)
N1—C6—C7—N2	1.9 (3)	O6—N4—C18—C19	15.1 (3)
C11—C6—C7—N2	−178.70 (19)	O5—N4—C18—C19	−162.99 (19)
O3—N2—C7—C8	14.5 (3)	O5—N4—C18—C19	−162.99 (19)
O2—N2—C7—C8	−163.90 (19)	O6—N4—C18—C17	−165.5 (2)
O2—N2—C7—C8	−163.90 (19)	O5—N4—C18—C17	16.5 (3)
O3—N2—C7—C6	−165.80 (19)	O5—N4—C18—C17	16.5 (3)
O2—N2—C7—C6	15.8 (3)	C17—C18—C19—C20	−0.1 (3)
O2—N2—C7—C6	15.8 (3)	N4—C18—C19—C20	179.4 (2)
C6—C7—C8—C9	−1.5 (3)	C18—C19—C20—C21	1.1 (3)
N2—C7—C8—C9	178.20 (19)	C19—C20—C21—C22	−1.0 (3)
C7—C8—C9—C10	0.8 (3)	C20—C21—C22—C17	−0.2 (3)
C8—C9—C10—C11	0.3 (3)	N3—C17—C22—C21	−177.4 (2)
C9—C10—C11—C6	−0.8 (3)	C18—C17—C22—C21	1.2 (3)

### Hydrogen-bond geometry (Å, º)

**Table:**

*D*—H···*A*	*D*—H	H···*A*	*D*···*A*	*D*—H···*A*
N1—H1*N*···O2	0.81 (3)	1.96 (3)	2.634 (2)	140 (3)
N3—H3*N*···O5	0.84 (2)	1.93 (2)	2.616 (2)	138 (2)
C1—H1···O4^i^	0.95	2.50	3.209 (3)	131
C8—H8···O5^ii^	0.95	2.52	3.248 (3)	133
C19—H19···O2^iii^	0.95	2.63	3.377 (3)	136
C11—H11···S2^iii^	0.95	2.92	3.732 (2)	144
C22—H22···S1^ii^	0.95	2.84	3.689 (2)	149
C12—H12···O1^iv^	0.95	2.47	3.198 (3)	133

Symmetry codes: (i) *x*, −*y*+3/2, *z*−1/2; (ii) *x*, −*y*+3/2, *z*+1/2; (iii) *x*−1, −*y*+3/2, *z*−1/2; (iv) *x*+1, −*y*+3/2, *z*+1/2.
